# 3D correlative light and electron microscopy of cultured cells using serial blockface scanning electron microscopy

**DOI:** 10.1242/jcs.188433

**Published:** 2017-01-01

**Authors:** Matthew R. G. Russell, Thomas R. Lerner, Jemima J. Burden, David O. Nkwe, Annegret Pelchen-Matthews, Marie-Charlotte Domart, Joanne Durgan, Anne Weston, Martin L. Jones, Christopher J. Peddie, Raffaella Carzaniga, Oliver Florey, Mark Marsh, Maximiliano G. Gutierrez, Lucy M. Collinson

**Affiliations:** 1 Electron Microscopy Science Technology Platform, The Francis Crick Institute, 1 Midland Road, London NW1 1AT, UK; 2 Host-pathogen Interactions in Tuberculosis Laboratory, The Francis Crick Institute, Mill Hill Laboratory, The Ridgeway, Mill Hill, London NW7 1AA, UK; 3 MRC Laboratory for Molecular Cell Biology, University College London, Gower Street, London WC1E 6BT, UK; 4 The Babraham Institute, Cambridge CB22 3AT, UK

**Keywords:** 3D correlative light and electron microscopy, Serial blockface SEM, *Mycobacterium tuberculosis*, Autophagy, HIV-1, Entosis

## Abstract

The processes of life take place in multiple dimensions, but imaging these processes in even three dimensions is challenging. Here, we describe a workflow for 3D correlative light and electron microscopy (CLEM) of cell monolayers using fluorescence microscopy to identify and follow biological events, combined with serial blockface scanning electron microscopy to analyse the underlying ultrastructure. The workflow encompasses all steps from cell culture to sample processing, imaging strategy, and 3D image processing and analysis. We demonstrate successful application of the workflow to three studies, each aiming to better understand complex and dynamic biological processes, including bacterial and viral infections of cultured cells and formation of entotic cell-in-cell structures commonly observed in tumours. Our workflow revealed new insight into the replicative niche of *Mycobacterium tuberculosis* in primary human lymphatic endothelial cells, HIV-1 in human monocyte-derived macrophages, and the composition of the entotic vacuole. The broad application of this 3D CLEM technique will make it a useful addition to the correlative imaging toolbox for biomedical research.

## Abstract (precis)

**Summary:** A new workflow for 3D correlative light and electron microscopy of cell monolayers, applied to studies of *M.-tuberculosis*-infected cells, HIV-1-infected cells and entotic cell-in-cell structures.

## INTRODUCTION

Correlative light and electron microscopy (CLEM) is a widely used technique that allows researchers to combine two separate imaging modalities in a manner that overcomes the limitations of each (Muller-Reichert and Verkade, 2014, 2012). Fluorescence microscopy allows identification of tagged macromolecules and analysis of their biological roles within living cells and tissues. However, information is limited by the resolution of the light microscope and by lack of fine structural detail elsewhere in the cell. Electron microscopy offers much-improved resolution and, crucially, ultrastructural context, but at the expense of imaging a fixed sample and with a restricted field of view. By combining these two techniques, CLEM makes it possible to target rare and/or dynamic bio-events for structural analysis at high resolution.

Electron microscopy for CLEM was traditionally performed by manually serial sectioning the sample and imaging each section using transmission electron microscopy (TEM), usually requiring more than 100 sections to image a single cell. However, automated systems based on the scanning electron microscopy (SEM) are gaining in popularity. These innovative ‘volume electron microscopy’ techniques (Kremer et al., 2015; Peddie and Collinson, 2014) include array tomography (Micheva and Smith, 2007; Wacker and Schroeder, 2013), focused ion beam SEM (FIB-SEM) (Heymann et al., 2006) and serial blockface SEM (SBF-SEM) (Denk and Horstmann, 2004). In array tomography, sections are cut manually or automatically (Hayworth et al., 2006) and placed in an array on a silicon wafer for large area imaging in the SEM. In FIB-SEM, a gallium ion beam sputters slices of material from the blockface, whereas in SBF-SEM a diamond knife in a miniaturised ultramicrotome removes thin slices from the blockface. In both cases, the revealed surface of the block is imaged using a backscattered electron (BSE) detector, and the process repeated sequentially to build up a stack of images through the volume of the sample.

Previous studies have reported the combination of CLEM workflows with volume electron microscopy techniques. Correlative or conjugate array tomography (CAT) has been applied to map synapses in brain tissue (Oberti et al., 2011; Collman et al., 2015); correlative light and FIB-SEM (Lucas et al., 2012, 2014) has been used to image cells (Murphy et al., 2011; Beckwith et al., 2015), developing blood vessels in zebrafish (Armer et al., 2009; Bushby et al., 2012) and dendritic spines and synapses in brain tissue (Maco et al., 2013; Bosch et al., 2015; Blazquez-Llorca et al., 2015). Here, we report a correlative workflow for 3D fluorescence microscopy to SBF-SEM, and demonstrate application to several different biological questions, particularly focusing on the geometry of cell monolayers.

The workflow is illustrated using our recent analysis of primary human lymphatic endothelial cells (hLECs) infected with *Mycobacterium tuberculosis* (Mtb), a newly identified niche for the bacterium in the lymph nodes of patients with tuberculosis (Lerner et al., 2016). In our study, we determined that there were fewer intracellular bacteria when the process of autophagy was inhibited. We hypothesised that the bacteria were growing in autophagosomes, and this was investigated using this 3D CLEM workflow. First, we identified lymphatic endothelial cells that had been transduced with LC3–RFP (the LC3B form, also known as MAP1LC3B) and also contained EGFP-expressing bacteria. Next, live imaging allowed us to track an infected cell over 5 days, by which time it was clear that the bacteria were alive, growing and dividing (the EGFP signal was increasing in area) despite being located in an LC3+ compartment, which is conventionally associated with Mtb killing. However, fluorescence microscopy did not have sufficient resolution to answer basic questions regarding the nature of the compartment, such as bacterial load, host and bacterial membrane structure, and internal composition of the LC3+ compartment. In addition, we could not be confident that the LC3+ compartment was a continuous structure completely encapsulating the bacteria in all axes.

We applied the same workflow to study entosis, an intriguing example of cell cannibalism in which one live epithelial cell is completely engulfed by another (Overholtzer et al., 2007; Overholtzer and Brugge, 2008). This process leads to the formation of ‘cell-in-cell’ structures, which are commonly observed in human cancers. Following engulfment, the internalised cell can remain viable for many hours, residing in a single membrane entotic vacuole formed by invagination of the host plasma membrane. The majority of internalised cells are ultimately killed and digested by their host through a process involving a non-canonical function for autophagy proteins and lysosomal degradation (Florey et al., 2011). Entosis is distinct from other types of macro-endocytic engulfment, such as phagocytosis, as the internalising cell plays an active role in its own uptake, dependent on adherens junctions and actinomyosin contractility (Overholtzer et al., 2007; Sun et al., 2014). In light of the differences between entosis and other well-studied forms of engulfment, and the difficulty in determining whether cells are fully engulfed using light microscopy, we sought to examine the cell-in-cell structures and the entotic vacuole in more detail using 3D CLEM.

Finally, we illustrate how the workflow was applied to a study of human monocyte-derived macrophages (MDMs) infected with human immunodeficiency virus type 1 (HIV-1) (Nkwe et al., 2016). HIV-1-infected MDMs accumulate large numbers of virus particles in intracellular plasma membrane-connected compartments (IPMCs) (Mlcochova et al., 2013; Deneka et al., 2007). This virus has been proposed to be long-lived and environmentally protected, sequestered away from the immune response of the host and possibly antiviral drugs (Sharova et al., 2005; Mlcochova et al., 2013). Although IPMCs have been shown to contain mature and immature virus particles, whether they are the main site of HIV assembly, a site of particle storage or a location where engulfed exogenous viruses can accumulate, has been a topic of considerable debate (Welsch et al., 2011; Marsh et al., 2009; Tan and Sattentau, 2013). Understanding the contribution and regulation of this compartment is therefore of great interest, especially as there is increasing evidence that macrophages play an important role in establishing infection *in vivo* (Sewald et al., 2015) and might also play a role in HIV-associated neurocognitive disorders in patients on antiretroviral therapy (Rappaport and Volsky, 2015). The highly pleomorphic structure of IPMCs was beyond the resolution of the light microscope, so we used our 3D CLEM workflow to identify a macrophage with a prominent IPMC and then imaged through the volume with sufficient resolution to clearly identify ultrastructural features.

## RESULTS

### A workflow for 3D CLEM using SBF-SEM

**Fig. 1.**
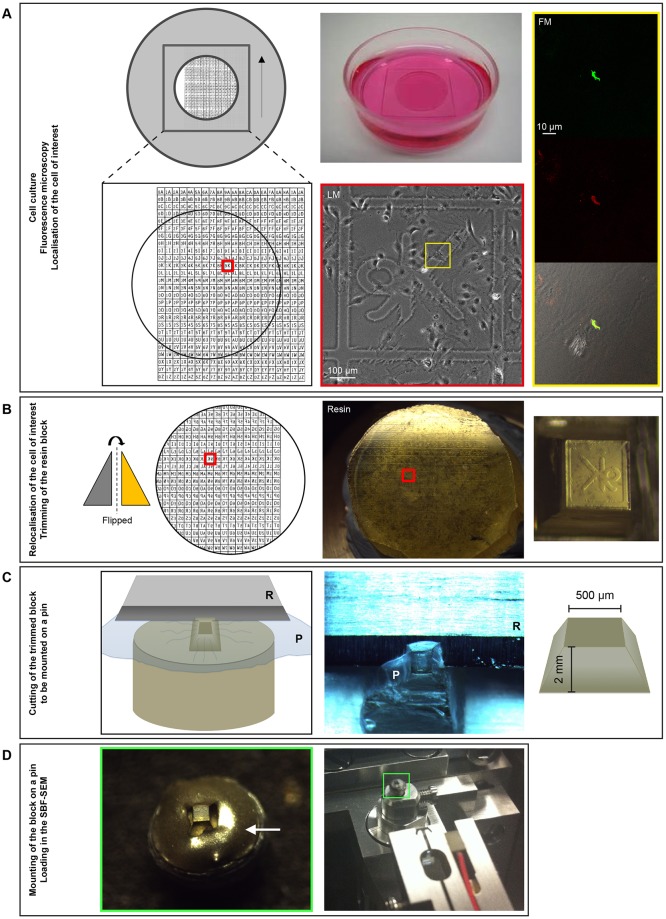
**Workflow for sample preparation from live-cell imaging to preparation of resin-embedded cells for SBF-SEM.** (A) Left panel: diagram of gridded glass-bottom dishes (MatTek Corp. # P35G-2-14-CGRD) with cell position indicated by red box. Middle panel: MatTek dish with live cells in culture medium (top) and brightfield image (bottom) of cell of interest (yellow inset) at grid coordinate 9K. Right panel: confocal fluorescence images were then acquired. (B) Following resin-embedding, the coverslip was removed, leaving an inverted cast of the grid on top of a monolayer of embedded cells. The cell of interest was relocated (red inset) and trimmed (right panel). (C) The frustum was covered with parafilm (P) and removed with a razorblade (R) (middle and right panel) (Movie 1). (D) This was mounted onto an aluminium pin using conductive epoxy glue (left panel, white arrow) (Movie 2), and secured in the SBF-SEM sample holder (right panel, green inset).

A substantial portion of life science research is performed using cells grown in culture. We developed a workflow for 3D correlative analysis of fluorescently labelled structures in cells cultured on glass coverslips (Fig. 1). The workflow was based on the classic pre-embedding CLEM method (Polishchuk et al., 2000) that moves from light microscopy to TEM, but with modifications to sample preparation and imaging strategies tailored to SBF-SEM.

### From live-cell imaging to resin-embedded cells

Cells were grown on photo-etched gridded glass-bottom dishes, with the coordinates used for recording regions of interest (ROI) (Fig. 1A). A diamond scorer or forceps can also be used to mark cell positions on plain glass coverslips. In general, cells were imaged using time-lapse confocal microscopy until the biological event occurred, at which point they were chemically fixed by addition of aldehydes to the cell medium. For confocal *z*-stacks, images were acquired from the whole volume of the cell starting at the coverslip, which was essential for successful 3D fluorescence microscopy and 3D electron microscopy correlation. A map of the cell location was then created using phase-contrast or brightfield light microscopy over a large field of view (FOV) to show cell shape, cell position in relation to surrounding cells, and the closest number and lines of the grid.

Fluorescence imaging of live (see Fig. 7) or fixed (see Figs 4–6,8) cells was used for correlation with SBF-SEM data, depending on the dynamics of the process being studied. Acquisition parameters varied between systems and were dependent on the experimental conditions and fluorophores used (Table S1). In general, we suggest using the highest magnification and highest NA objective available (at least 1024×1024 pixels, zoom 2, line averaging 4, minimal *z*-section thickness and *z*-section interval corresponding to half the *z*-section thickness). Markers for endoplasmic reticulum (ER), mitochondria and lysosomes can be added to improve fluorescence microscopy and electron microscopy data alignment, by increasing the number of landmarks.

Following primary fixation, cells were processed into resin using the method of Deerinck and Ellisman (Deerinck et al., 2010), which adds extra heavy metal into the cells for improved conductivity during imaging. Removal of the coverslip from the polymerised resin block with liquid nitrogen resulted in a monolayer of cells at the blockface, overlaid by a positive cast of the grid (Fig. 1B). The ROI was trimmed so that the cell of interest was in the centre of the blockface (Fig. 1B), and cut from the block to yield a frustum (truncated square pyramid) of 2-mm high with a face of ∼500×500 µm (Fig. 1C; Movie 1). The released frustum was mounted onto an aluminium pin using conductive epoxy glue to aid in charge dissipation during imaging (Fig. 1D; Movie 2). The block was mounted with a slight tilt so that the face was ∼5° from horizontal to aid the approach to the ROI.

### Approach strategy in the SBF-SEM

**Fig. 2.**
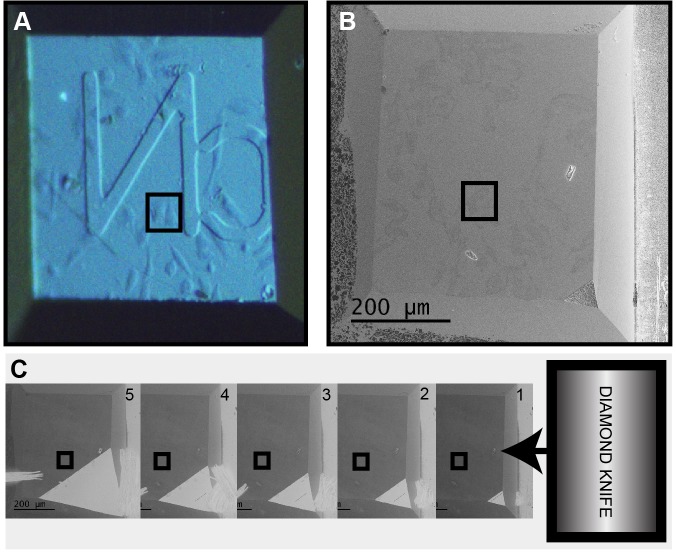
**Approach strategy in the SBF-SEM.** (A) The cell of interest (black box) was relocated at the blockface. (B) The cells were visible through the Pt layer when imaging at 5 kV using the BSE detector. (C) The diamond knife was aligned to the highest point of the blockface and the coarse approach performed at 100-nm slice thickness.

The block was sputter-coated with a thin layer of platinum (Pt) to further aid charge dissipation. The pin was secured in the sample holder and the cell of interest identified (Fig. 2A). The diamond knife was aligned parallel to the front edge of the blockface and the height adjusted to the highest corner or edge of the blockface. The slight tilt applied to the blockface during mounting allowed us to approach the cell of interest from one side, so that cutting and imaging conditions were stable prior to starting the imaging run. The coarse approach to the cell was performed with the chamber door open, at 100-nm slice thickness, after which the door was closed and the chamber pumped to ∼5 Pa. The cell of interest was relocated at 5 kV using the BSE detector (Fig. 2B), and a fine approach performed using 50-nm cuts (Fig. 2C). Imaging and cutting conditions were optimised on an adjacent cell before setting up the SBF-SEM run.

### Imaging strategy

Imaging cell monolayers in the SBF-SEM was challenging because of the high proportion of insulating resin. Conductive paths through the sample were therefore few or absent, leading to a build-up of electrons at the sample surface and charging artefacts. Managing electron dose and charging at the specimen surface was therefore crucial for acquiring high-quality data. Charging artefacts were mitigated by balancing a number of inter-dependent imaging parameters. In general, we used a high-beam current setting to generate sufficient BSE signal, while imaging at low voltage (which also helps limit imaging depth), at a vacuum pressure of ∼5–10 Pa [which suppresses charging but worsens the signal-to-noise ratio (SNR)], in combination with a fast per-pixel dwell time, and use of a small final aperture (Table S2).

**Fig. 3.**
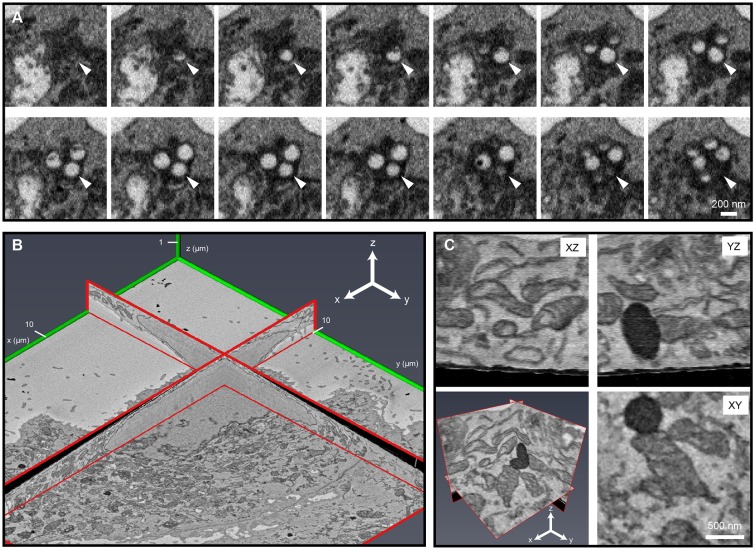
**Demonstration of stable ultrathin cutting in the SBF-SEM.** (A) SBF-SEM of mouse MD4 B cells containing antigen-coated 200-nm beads (Thaunat et al., 2012) cut at 15-nm section thickness and imaged at a pixel size of 15 nm. One of the two bead clusters quantified is shown in 14 consecutive slices (white arrows). (B) SBF-SEM of Vero E6 cells cut at 10-nm section thickness and imaged at a pixel size of 10 nm (Movie 3). (C) Panels show orthoslices from a 2×2×2 μm cube (bottom left panel) extracted from the dataset shown in B, demonstrating isotropic voxel resolution throughout the volume.

For successful serial imaging, it was crucial to balance signal generation with reduced charging and stable cutting. When the electron dose was too high, sectioning artefacts, including inconsistent cutting and rippling at the resin surface, were visible in empty resin, even when areas containing cellular material continued to section well. Generally, increased section thicknesses tolerated higher electron doses better, at the expense of axial resolution. Under optimal conditions, it was possible to attain an axial resolution of 10–15 nm (Fig. 3). We demonstrated stable serial imaging at 15-nm section thickness over 1000 slices on a resin block containing B-cells loaded with 200-nm beads (Thaunat et al., 2012). As expected, it took ∼13 cuts (mean±s.e.m., 13.1±0.42, *n*=12 beads from two separate clusters) to section through a single 200-nm bead (Fig. 3A). We also achieved stable serial imaging at a 10-nm section thickness over 370 slices on a resin block containing Vero E6 cells (Fig. 3B; Movie 3).

### Overlaying 3D light microscopy and 3D electron microscopy data

A typical volume electron microscopy dataset consisting of hundreds of slices acquired at 8192×8192 pixels can reach well beyond 100 GB. Visualising and processing ‘big data’ can become a significant problem. We mitigated this by separating the overlay process into two distinct stages: calculation of the spatial transform using binned images, and subsequent application of the transform to the full resolution images.

In aligning two 3D datasets from different imaging modalities, a six-axis alignment must be considered (*x*, *y*, *z*, pitch, yaw, roll), in addition to scaling and shear. Processing of the sample between light microscopy and electron microscopy imaging can also introduce non-linear deformations, which cannot be accounted for with a simple affine transformation. To help correct such effects, we used the BigWarp plugin in Fiji (http://fiji.sc/BigWarp; BigWarp version 2.1.0, Fiji based on ImageJ 1.50e on Windows 10), which harnesses the BigDataViewer system (Pietzsch et al., 2015) allowing efficient handling and display of very large datasets. This is currently the only software that we are aware of that can do such an efficient alignment of two raw 3D datasets, including live transformation as landmarks are repositioned. BigWarp uses a thin-plate spline method with manual landmarks to map one dataset onto another, with the major advantage that the transform is encoded as a small text file containing just the landmark positions. A Fiji script (see https://figshare.com/s/33a422c43fde70ac8580) was written to generate an image stack showing BigWarp landmark locations in 3D. Given that the transformation is mathematically well-defined for a given set of landmarks, anyone with access to the raw data files can use the landmarks to immediately view the transformed data and, if necessary, adjust the transform without having to rewrite any image files.

### Proof of principle 1 – understanding Mtb environments during intracellular growth

**Fig. 4.**
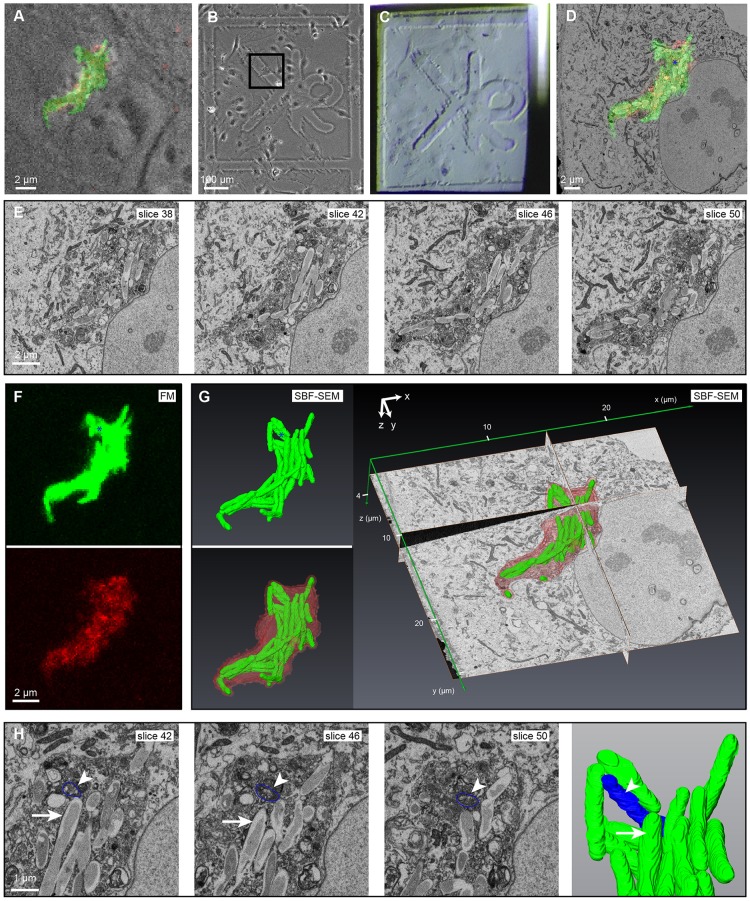
**3D CLEM reveals the subcellular location of Mtb in lymphatic endothelial cells.** (A–G) Data from Lerner et al., 2016 is shown to illustrate in detail the workflow used to acquire this data. (A) Overlay of GFP–Mtb (green) and LC3–RFP (red) signals onto a phase-contrast image of the infected cell. (B) Location map of the cell of interest. (C) The grid was transferred to the blockface during embedding, and the block trimmed and mounted for SBF-SEM. The coarse approach cuts have started to clean away the Pt-coat at the right side of the block. (D) Coarse overlay of the fluorescence microscopy onto an SBF-SEM image mid-run to confirm the structure of interest is in the FOV. (E) SBF-SEM images through the Mtb colony. (G) SBF-SEM segmentation and reconstruction; the Mtb (green) and perimeter membrane (red) were segmented and modelled with reference to the coarse overlay (D) and fluorescence microscopy (F, maximum intensity projections). (H) Electron-dense bacterium segmented in blue (arrowhead in H and asterisk in D,F,G) compared with an electron-lucent bacterium (arrow).

**Fig. 5.**
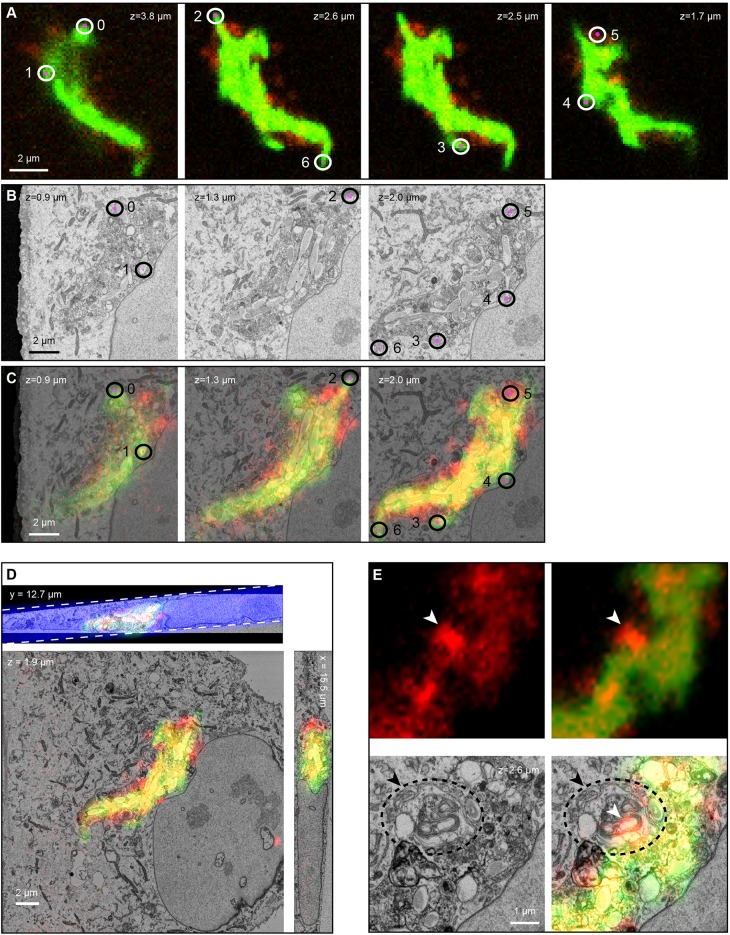
**Fine alignment of 3D light microscopy and 3D electron microscopy data using BigDataViewer and BigWarp.** New analysis of the dataset used for Lerner et al., 2016. Landmarks were placed (A–C) to generate the fine alignment (D), which could then be used to identify an LC3+ membrane whorl (E). (A) Raw fluorescence slices, with GFP–Mtb (green) and LC3–RFP (red). (B) SBF-SEM slices. (C) Overlay after BigWarp transformation of the fluorescence stack using the landmarks shown. The *xz* plane (top panel) shows the tilt of the Pt layer (equating to the coverslip) with respect to the horizontal plane of the SBF-SEM data. The inclined blue rectangle demarcates a slice through the phase-contrast image volume, showing its coplanarity with the Pt layer. (E) Membrane-rich LC3+ whorl. A–C, E and the *xz* and *zy* planes of D are snapshots of the BigWarp graphical user interface, with annotations, except for the magenta dots, added later.

**Fig. 6.**
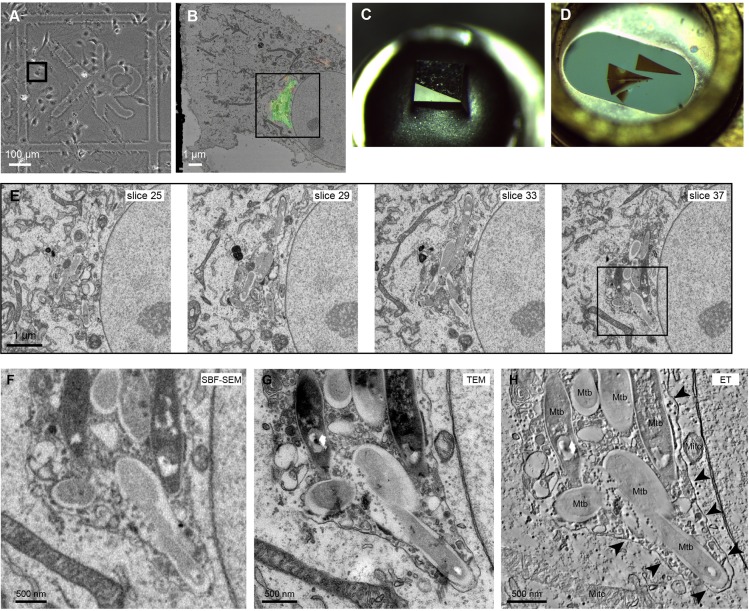
**Complementing SBF-SEM with high-resolution TEM images of the same structure.** (A) Location map of the cell of interest, and (B) coarse overlay of the fluorescence microscopy onto an SBF-SEM image mid-run to confirm the structure of interest is in the FOV. (C) The block was then removed from the SBF-SEM, and (D) serial sections collected for TEM on a formvar-coated slot grid. Using this technique, it was possible to follow the same structure from (E,F) SBF-SEM to (G) TEM. (H) High-resolution images were acquired by electron tomography (ET), showing the single perimeter membrane (black arrowheads) and individual bacilli (Mtb) within the compartment. Mito, mitochondria.

As part of a recent study, we showed that disrupting autophagy inhibited Mtb growth in resting human endothelial lymphocytes. We used fluorescence microscopy to identify and follow Mtb-infected cells, and correlated this data with SBF-SEM to reveal that Mtb grew inside the autophagosomes in which they were completely encapsulated. SBF-SEM allowed us to observe the full cellular microenvironment in which Mtb colonies grew including the compartments in which they were located and the host organelles that interact with them (Lerner et al., 2016). Here, we describe in detail the workflow used to obtain this information, and further analyse the dataset from this cell to reveal new biological information (Figs 4 and 5). We also describe an extended workflow, linking SBF-SEM to TEM, to reveal higher resolution ultrastructural detail from an LC3+ Mtb-compartment in another cell of interest (Fig. 6).

Imaging the BSL3 pathogen Mtb required carefully considered and verified safety protocols, and specialised equipment, all of which must be contained within a dedicated facility. Our BSL3 laboratory has a confocal microscope equipped with an environmental chamber that enabled live-cell imaging of eukaryotic cells infected with Mtb over extended periods of time (routinely up to 12 days), necessary due to the slow growth of the pathogen. hLECs expressing the autophagosomal marker LC3–RFP were infected with EGFP-expressing Mtb and imaged until colocalisation of the bacteria with LC3 was observed (Fig. 4A). The cell of interest was followed through sample preparation for SBF-SEM as described (Fig. 4B,C) and serial images acquired (Fig. 4E). Coarse overlay of fluorescence microscopy and SBF-SEM images (Fig. 4D) allowed us to identify individual mycobacteria in a group within the target cell, bound by a perimeter membrane (Fig. 4E). The structural resolution of the images was sufficient to allow segmentation of the Mtb and the surrounding host membrane (Fig. 4G). Importantly, 3D CLEM identified morphologically distinct bacteria that were missed in the initial segmentation because they were more electron-dense than other bacteria in the colony (Fig. 4D,F–H, marked in blue). To refine and extend the analysis beyond that shown in Lerner et al., 2016, 3D fluorescence microscopy and electron microscopy image overlays were created using BigDataViewer and BigWarp (Fig. 5D), using Mtb and LC3+ perimeter membranes as landmarks (Fig. 5A–C; Table S5). This precise overlay identified additional LC3+ membrane whorls inside the compartment that resembled lysosomes or autolysosomes (Fig. 5E). We performed new analysis on the resulting models and found that the average Mtb volume was 0.44 µm^3^ (*n*=39) (Table S3), consistent with previously published 2D dimensions, suggesting that there is no growth defect (Cook et al., 2009).

LC3 is known to associate with various compartments, including canonical double-membrane autophagosomes (Mizushima et al., 2002) and non-canonical single-membrane compartments (Sprenkeler et al., 2016). To definitively distinguish single- and double-membranes, and improve our understanding of the LC3+ compartment in which Mtb grow, we introduced a transfer step from SBF-SEM to higher resolution TEM. A new cell of interest was selected and imaged by 3D CLEM, and a coarse overlay of the fluorescence microscopy and electron microscopy images performed, which identified a group of bacilli in another LC3+ compartment (Fig. 6A,B). The SBF-SEM run was stopped ∼30 sections into the compartment. The pin was removed and placed in a standard ultramicrotome. Given that the blockface was already polished to a flat surface, having been cut in the SBF-SEM (Fig. 6C), it was possible to cut complete sections within one or two cuts (Fig. 6D). The sample was already highly contrasted by the *en bloc* stain, so no post-staining of the TEM sections was required. It was thus possible to inspect a single bacterium in adjacent SBF-SEM and TEM sections (Fig. 6F,G). Electron tomography was performed on the TEM sections to achieve the highest possible resolution, and unequivocally identify a single limiting membrane surrounding the group of bacteria (Fig. 6H), demonstrating that this particular compartment was not a double-membrane-bound autophagosome.

### Proof of principle 2 – visualising cell-in-cell structures formed by entosis

**Fig. 7.**
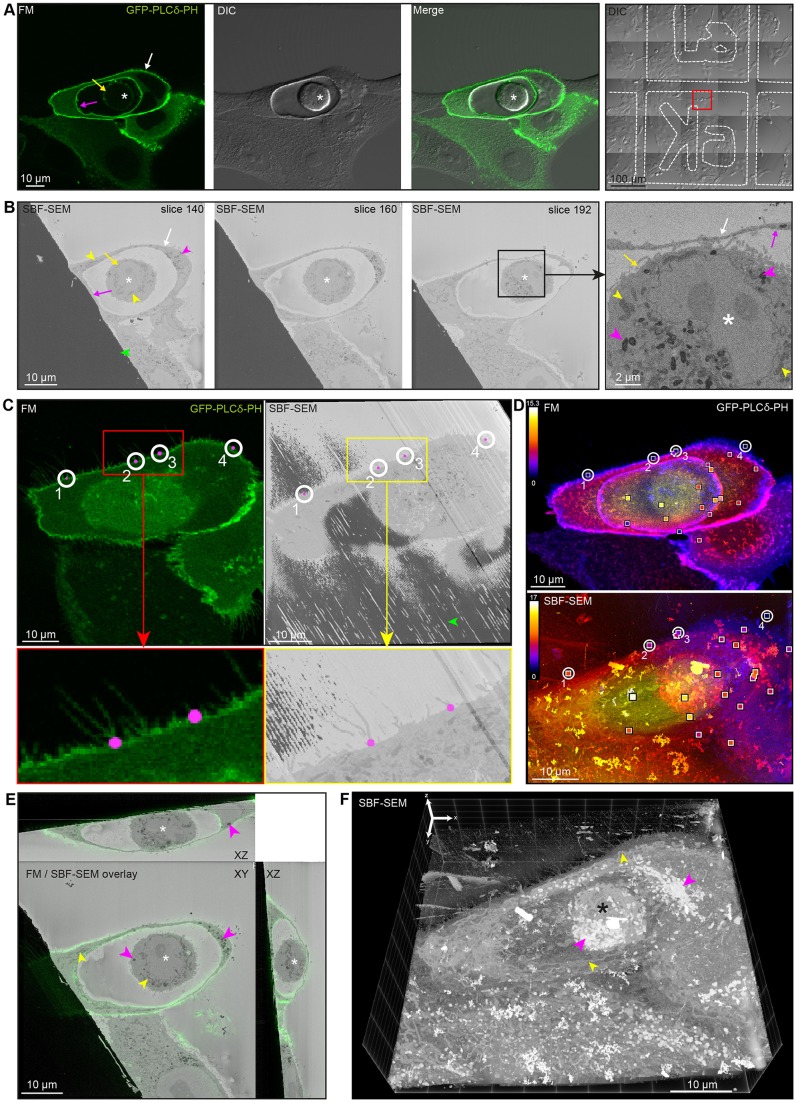
**3D CLEM of a cell-in-cell structure formed by entosis.** (A) MCF10A breast epithelial cells were transfected with GFP–PLCδ-PH (green), which is expressed at both the host and engulfed cell plasma membranes and the entotic vacuole. A cell of interest (red box) was identified in live-cell conditions. (B) SBF-SEM images through the entosed cell (Movie 4). (C) Fluorescence microscopy (FM) and SBF-SEM data were aligned using BigWarp. The GFP–PLC∂-PH image (left) and oblique slice through the electron microscopy dataset (right) show the position of four landmarks using filopodia at the bottom of the cell (insets). (D) In total, 21 landmarks were selected on the plasma membrane of the cells and entotic vacuole (Movie 5) shown in depth-coded maximum intensity projections (top, GFP–PLC∂-PH; bottom, SBF-SEM). Pixel colour denotes the *z* position at which the maximum intensity in the stack is found. The landmarks are shown as white squares with an inset colour corresponding to the depth position of the landmark. The colour bar indicates the depth in µm. (E) Overlay of the resliced GFP–PLC∂-PH (green) image stack and the SBF-SEM stack using BigWarp, shown in all three orthoslices. (F) 3D reconstruction of the SBF-SEM stack showing the entotic cell within its host cell. The SBF-SEM image was inverted and the Pt signal subtracted to aid visualisation in 3D (Movie 5). Asterisk, entotic cell; white arrow, host cell plasma membrane; yellow arrow, engulfed cell plasma membrane; magenta arrow, entotic vacuole; yellow arrowhead, mitochondria; magenta arrowhead, lipid vacuole; green arrowhead, Pt.

To study entosis by 3D CLEM, MCF10A breast epithelial cells were transfected with the phospholipid phosphatidylinositol 4,5-bisphosphate [PI(4,5)P_2_] sensor, as a live marker of the plasma membrane, and seeded onto gridded glass-bottom dishes where cell-in-cell structures formed. Unlike phagocytosis, where PI(4,5)P_2_ is lost from phagosomes soon after formation (Botelho et al., 2000), we observed that PI(4,5)P_2_ remains enriched at both the host and engulfed cell plasma membranes, and the entotic vacuole (Fig. 7A). Apparent differences in intensity between membranes most likely relate to variable expression levels of the sensor in the cell population rather than PI(4,5)P_2_ levels. The entotic cell position was recorded using grid maps taken with differential interference contrast (DIC) in live-cell conditions. By SBF-SEM, the internalised cell appeared morphologically viable with an intact plasma membrane and nucleus, confirming this as a live-cell engulfment event (Fig. 7B; Movie 4). Fluorescence microscopy and SBF-SEM images were aligned using BigWarp. The first landmarks were placed at the bottom of the cell, at the base of the filopodia (Fig. 7C). A total of 21 landmarks were selected on the plasma membrane of the cells and the entotic vacuole throughout the volume (Fig. 7D; Table S6, Movie 5). The overlaid fluorescence microscopy and SBF-SEM data confirmed that the entotic vacuole appeared similar in characteristics to plasma membrane (Fig. 7E), consistent with retention of PI(4,5)P_2_. 3D CLEM confirmed that the entotic cell was completely engulfed by the host cell (Fig. 7F; Movie 5), which has not been previously observed. Given the limitations of light microscopy in observing and differentiating between the vacuole and plasma membranes, and the possibility of missing gaps through the whole cell volume using 2D electron microscopy, 3D CLEM was the only method that can confirm complete engulfment during entosis.

### Proof of principle 3 – insights into HIV trafficking in human MDMs

**Fig. 8.**
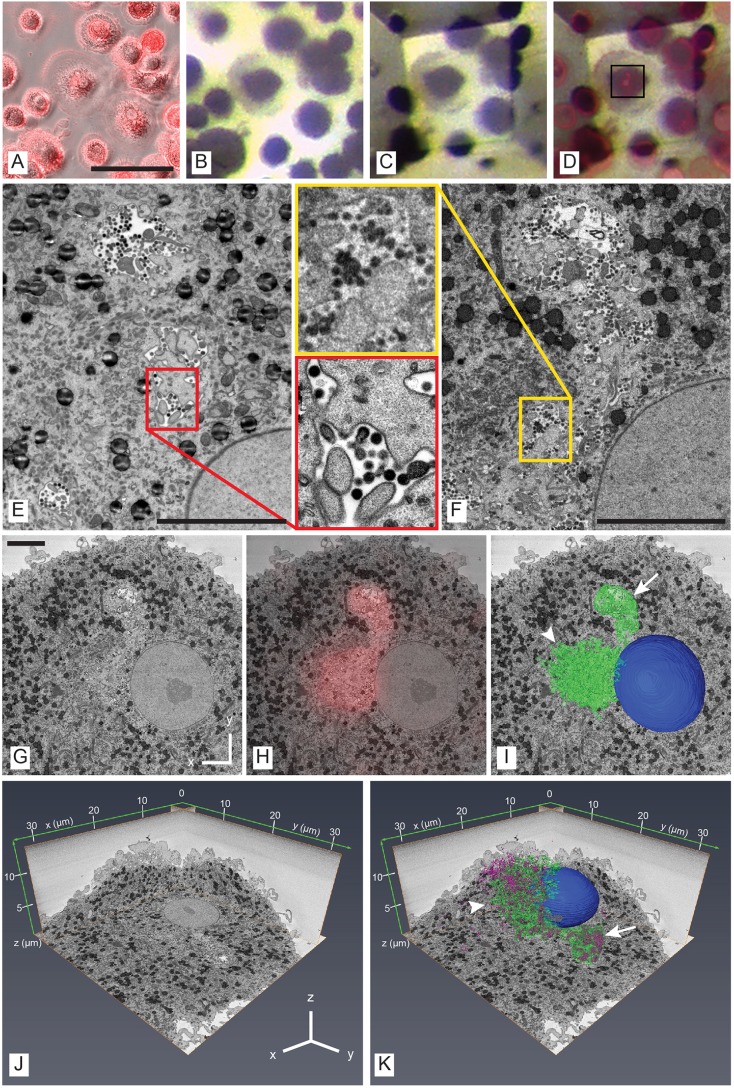
**3D CLEM of HIV-1-infected MDMs.** MDMs were infected with budding-arrested HIV-1 mutant virus (Nkwe et al., 2016) and cells with prominent IPMCs were identified by enrichment of CellMask™ labelling. (A) Widefield fluorescence microscopy of IPMC labelling overlaid over a phase-contrast image of the cell of interest. Scale bar: 100 µm. (B) Resin blockface with cell of interest after processing for SBF-SEM, trimmed (C) and overlaid with fluorescence microscopy (D). (E) TEM of infected cell. Scale bar: 5 µm. (F) SBF-SEM of matching region (*x*,*y*). Scale bar: 5 µm. (G) SBF-SEM image of cell of interest. Scale bar: 5 µm. (H) CellMask™ widefield fluorescence microscopy image overlaid onto SBF-SEM image. (I) 3D reconstruction of IPMC (green) and nucleus (blue) overlaid onto SBF-SEM image. (J) *xy*, *xz* and *yz* orthoslices from SBF-SEM data. (K) 3D reconstructions of arrested HIV-1 assembly sites (purple), the IPMC (green) and the nucleus (blue) projected over xy, xz and yz orthoslices from SBF-SEM data. In I and K, arrow, compact domain; arrowhead, convoluted domain.

To identify and localise MDMs infected with budding-arrested HIV-1 mutants with prominent IPMCs, we used the live-cell stain CellMask™ Orange, a fluorescent plasma membrane dye that accesses IPMCs through surface connections (Fig. 8A) (Mlcochova et al., 2013). The cell of interest was relocated in the resin block using the maps and fluorescence microscopy data (Fig. 8B), trimmed (Fig. 8C,D) and the first few serial ultrathin sections collected to assess the quality of the sample and confirm the presence of viral buds by TEM (Fig. 8E and magnified insert). The sample was then transferred to the SBF-SEM and >300 images were collected. Although SBF-SEM images (Fig. 8F and magnified insert) were lower resolution than the TEM images (Fig. 8E and magnified insert), they had sufficient resolution over a large volume to segment and model the IPMC (whose location was confirmed by coarse overlay of 2D fluorescence microscopy data; BigWarp was not used in this case), revealing an intricate network of interconnected membranes (Fig. 8G–I). Manual segmentation of the virus profiles enabled 3D localisation and visualisation of the assembling particles with respect to the IPMC (Fig. 8J,K) and cell surface proper (Nkwe et al., 2016). The CellMask™ staining indicated that the IPMC was comprised of two linked domains with distinct morphologies: one compact domain that opened up to the cell surface through a single narrow channel (Fig. 8I,K; arrow), and a second domain comprising a vast interconnected mesh of channels, with virions present throughout, that had multiple openings to the cell surface (Fig. 8I,K; arrowhead). The two domains were linked by multiple channels of varying diameter, each narrowing at points to less than 100 nm (Fig. 8H,I) (Nkwe et al., 2016). Further quantitative analysis of the 3D model for this paper showed that the two domains not only differ in morphology but also in size and viral contribution (Table S4). More specifically, the domain displaying an interconnected meshwork morphology contributes more than five times the area of IPMC membrane when compared to the compact domain, but contains only 1.8 times the number of virus buds, indicating a threefold enrichment of budding virus profiles in the compact IPMC domain.

## DISCUSSION

We present a workflow for 3D CLEM, from fluorescence microscopy to SBF-SEM, applied to analyse a range of biological questions that use a monolayer of cultured cells as a model system. At an imaging rate of one cell per day, CLEM analysis starts to become routine and quantitative. When lateral resolution is limiting, for example when the lipid bilayer of a biological membrane must be resolved, we have shown that serial sections can be taken from the blockface during the SBF-SEM run for TEM or electron tomography of the same structure.

Our workflow differs from those designed for correlative light and FIB-SEM (Karreman et al., 2016; Beckwith et al., 2015) because the sample geometry, cutting and imaging conditions differ between the two systems. The FIB-SEM is targeted, capable of milling very thin slices in the order of ∼5 nm, and allows multiple ROIs to be imaged at the blockface with less-severe charging artefacts. The SBF-SEM is simpler to set up and cuts faster over a larger area, but slice thickness is routinely set at 25–50 nm and the serial imaging run is more sensitive to charging artefacts (Peddie and Collinson, 2014). However, both techniques are destructive, which is an important consideration because CLEM experiments usually involve ‘single-shot’ specimens.

In the first study presented here, 3D CLEM revealed new information about the nature of the LC3+ compartment containing Mtb in hLECs (Lerner et al., 2016). New analysis of the original data allowed us to quantify the number of Mtb cells in the compartment more accurately, demonstrating that 3D CLEM was essential to identify morphologically distinct bacteria. Further quantitative analysis of the compartment revealed that average Mtb volume was normal, supporting our previous finding that these compartments promote bacterial growth rather than restrict it (Lerner et al., 2016). Future measurement of intracellular Mtb volume using this method could also help to assess the effect of antibiotics, given that they are known to affect both bacterial size and morphology (Farnia et al., 2010). We also analysed the content of the Mtb-containing compartment and found additional LC3+ structures that could influence bacterial growth, with morphologies reminiscent of lysosomes or autolysosomes. Analysing a second cell with our new 3D CLEM-TEM workflow, we also unequivocally show an Mtb-containing compartment surrounded by a single LC3+ membrane, also suggestive of an autolysosome or a non-canonical autophagosome.

3D CLEM also provided the first 3D ultrastructural analysis of the large and unusual cell-in-cell structures formed through entosis. Entosis is triggered by loss of matrix attachment, through a recently described engulfment mechanism, and is prevalent in human tumours. The internalised cell is held in a vacuole inside the host cytoplasm that, in an interesting parallel with the Mtb compartment, comprises a single membrane, which becomes LC3+ (Florey et al., 2011). Previously, it was not possible to determine whether live entotic cells were fully engulfed, leaving open the possibility that their persistent viability was perhaps due to incomplete internalisation. 3D CLEM has provided the required resolution to distinguish vacuole and plasma membrane through the whole cell volume and allows us to conclude that live cells can be held inside a fully scissioned vacuole. These data also yield new insights into phosphoinositide behaviour during entosis, suggesting that the profile of lipid changes differ in entosis from those in other macro-scale engulfment processes. Unlike phagosomes, which mature quickly upon formation and fuse with lysosomes, the entotic vacuole retains PI(4,5)P_2_ even after scission. Taken together, these data reveal the existence of an unusual intracellular PI(4,5)P_2_-positive vacuole compartment. The retention of PI(4,5)P_2_ and delay in maturation might explain why the internalised cell is able to remain viable even after engulfment and opens up a new line of research into the unique phospholipid dynamics associated with this important live-cell engulfment event.

3D CLEM of budding-arrested HIV-1-infected MDMs was perhaps the most challenging of the three proof-of-principle studies, requiring sufficient resolution to detect and trace individual virus budding intermediates over a whole-cell volume. The resulting data allowed high-resolution mapping of virus assembly sites and plasma membrane within an IPMC and a full 3D visualisation of this complex compartment. This 3D data was particularly interesting as it was not only in agreement with our previous 2D TEM ‘snapshots’ of the compartment (Pelchen-Matthews et al., 2012; Mlcochova et al., 2013; Deneka et al., 2007; Nkwe et al., 2016), but also allowed us to understand how the various morphologies previously described from 2D images of different cells might be connected in a single compartment with morphologically distinct domains. This work extends that reported in Nkwe et al. (2016) by quantifying virus load in the different IPMC domains, indicating a threefold enrichment of budding virus profiles in the compact domain, the consequences of which will be investigated in ongoing work. In summary, the mapping of virus assembly sites has enabled us to unequivocally demonstrate that the IPMC is a major site of HIV-1 assembly (Nkwe et al., 2016). This workflow provides a robust experimental approach for further investigations into relatively large, intricate and morphologically complex IPMCs and to assess their contribution to HIV assembly in macrophages.

Importantly, all three proof of principle studies illustrate the richness of 3D electron microscopy datasets, and show how a single dataset can be ‘mined’ multiple times for biological information. Following publication of the primary study, and deposition of the raw dataset in a public archive such as EMPIAR (Iudin et al., 2016), any scientist will be able to re-analyse the same data to extract further information about the same biological process (as shown here) or even about other biological processes occurring in the same volume. Working in this way will maximise the output from and impact of existing data resources, avoid large-scale expensive duplication of effort in data collection (especially when dealing with precious animal models), centralise multiple datasets collected by different groups from the same biological system to assess reproducibility, build a central resource of 3D datasets that will aid development of image analysis tools, and thus accelerate biomedical discovery.

## MATERIALS AND METHODS

### Cell culture – section thickness demonstrations

200-nm microspheres (Invitrogen) coated with antigen were loaded into mouse MD4 B cells as described in Thaunat et al. (2012). Vero E6 cells were cultured as described in Limpens et al. (2011).

### Cell culture and light microscopy – Mtb-infected cells

The full procedure for Mtb-infected cells was described in Lerner et al. (2016). Briefly, primary hLECs taken from inguinal lymph nodes (ScienCell Research Laboratories, #2500), were seeded in fibronectin-treated 35-mm glass-bottom dishes (e.g. MatTek Corp., USA, # P35G-2-14-CGRD or P35G-2/1.5-14-CGRD or Ibidi µ-Dish^35 mm, high^ Glass Bottom Grid-500) at a density of 30–50% confluence (∼10,000 cells) in 500 μl endothelial cell medium (ECM; ScienCell Research Laboratories, #1001) supplemented with 1% (v/v) endothelial cell growth supplement (ECGS; ScienCell Research Laboratories, #1052) and 5% (v/v) fetal bovine serum (FBS; ScienCell Research Laboratories, #0025) at 37°C with 5% CO_2_. While still in suspension, 10 μl Premo Autophagy Sensor LC3B–RFP BacMam 2.0 (Life Technologies, #P36236) was added to transduce the cells prior to overnight incubation at 37°C with 5% CO_2_. hLECs were then infected with *Mycobacterium tuberculosis* H37Rv constitutively expressing EGFP (as described in Lerner et al., 2016). After 5 h of infection at a multiplicity of infection of ten bacteria per cell, the MatTek dish was washed three times with PBS and 1 ml ECM was added containing 10 μl BacMam 2.0 LC3B–RFP to boost transduction; at this point the cells were ready for live-cell imaging.

The MatTek dish was securely fastened into a custom-made dish holder (in accordance with BSL3 regulations) and put onto the stage of a Leica SP5 AOBS Laser Scanning Confocal Microscope (Leica Microsystems, Germany) with an environmental control chamber (EMBLEM, Germany). Immersion oil used was Cargille Type 37 (Cargille Labs). Images were acquired using scanning mode *xyzt* with sequential acquisition (see Table S1 for full parameters). Frames were taken every 2 h for the first 12 h, then every 30 min. *z*-stacks of five slices were taken at each time point. Imaging continued for 121.5 h, until Mtb growth in an LC3+ compartment was clear.

The cells were fixed by addition of 4% PFA and 2.5% glutaraldehyde in 0.1 M phosphate buffer (pH 7.4) for 24 h at 4°C (a long fixation is necessary for BSL3 regulations). The fixative was then replaced with 0.1 M PB pH 7.4 and the cell of interest was re-located on the microscope using a 10× objective to find the grid-reference and then imaged again at high magnification (63×) to gain the final images to correlate with the electron microscopy images.

### Cell culture and light microscopy – entotic cells

MCF10A cells were cultured in DMEM/F12 (Gibco, 11320-033) supplemented with 5% horse serum (Gibco, 16050-122), 20 ng/ml EGF (Peprotech, AF-100-15), 10 µg/ml insulin (Sigma I9278), 0.5 µg/ml hydrocortisone (Sigma, H0888), 100 ng/µl cholera toxin (Sigma, C8052) and 100 U/ml penicillin and 100 µg/ml streptomycin (Gibco 15140-122). Cells were transfected with the GFP–PLCδ-PH containing plasmid (Addgene 21179) using a Nucleofector II instrument (Lonza) and Lonza nucleofection kit V (Lonza, VCA-1003) with programme T-024, following the manufacturer's guidelines, and cultured for 1 day. Transfected cells were re-seeded onto 35-mm gridded glass-bottom dishes (MatTek Corp., # P35G-2-14-CGRD) and used the following day.

Live-cell images were acquired with a Confocal Zeiss LSM 780 microscope (Carl Zeiss Ltd) at 37°C in a temperature- and CO_2_-controlled chamber, using Zen software (Carl Zeiss Ltd) (see Table S1 for full parameters). Cells were fixed by adding 8% formaldehyde (Sigma F8775) in 0.2 M phosphate buffer pH 7.4, in a 1:1 ratio with culture medium in the dish. Samples were further fixed in 2.5% glutaraldehyde (Sigma G5882) and 4% formaldehyde in 0.1 M phosphate buffer pH 7.4 for 30 min at room temperature. Samples were then kept in 1% formaldehyde in 0.1 M phosphate buffer pH 7.4 and stored at 4°C until processing.

### Cell culture and light microscopy – HIV-infected cells

A detailed description of the materials and methods for HIV-1 infected MDMs can be found in Nkwe et al. (2016). Briefly, stocks of infectious budding-arrested HIV-1 were prepared by transfecting HEK 293T cells, using FuGENE HD (Roche), with a mixture of mutant virus plasmid and pCMVGag WT. 7-day-old MDMs, grown on coverslips in 24-well plates were infected by spinoculation (centrifugation at 1300 ***g*** and 25°C for 2 h) with the rescued mutant virus at six focus-forming units per cell (spinoculation is not possible with the 35-mm photo-etched gridded glass-bottom dishes in the BSL3 environment). MDMs were cultured for 7 more days in Roswell Park Memorial Institute (RPMI) 1640 medium supplemented with 10% human serum, 100 units/ml penicillin, 100 µg/ml streptomycin, at 37°C and 5% CO_2_. Infected MDMs were incubated with 5 µg/ml CellMask™ Orange (Invitrogen Molecular Probes) for 10 min at 37°C before fixation with 4% formaldehyde. Cells were washed in PBS and imaged with a 20× objective on an inverted light microscope (Leica DMIL LED, Leica, Vienna, Austria) to localise cells with prominent IPMCs (see Table S1 for full parameters). Once identified, the cell of interest and its neighbours were marked by removing a ring of cells surrounding them using forceps. Further fixation was carried out with 2% formaldehyde and 1.5% glutaraldehyde in 0.1 M sodium cacodylate, prior to further processing.

### Sample preparation for electron microscopy

Samples were embedded using a protocol adapted from the NCMIR protocol (Deerinck et al., 2010). Briefly, after fixation, the samples were post-fixed in 2% osmium tetroxide and 1.5% potassium ferricyanide (v/v) for 1 h on ice, incubated in 1% thiocarbohydrazide in dH_2_O (w/v) for 20 min, followed by 2% osmium tetroxide in dH_2_O (w/v) for 30 min, and then washed in dH_2_O and incubated overnight in 1% aqueous uranyl acetate at 4°C. Cells were then stained with Walton's lead aspartate for 30 min at 60°C. The coverslips were removed from the dishes after submerging the bottom in methanol for 20 min to soften the glue. The cells were then dehydrated stepwise through an ethanol series on ice, incubated in a tin foil container in a 1:1 propylene oxide and Durcupan resin mixture, and embedded in Durcupan ACM^®^ resin according to the manufacturer's instructions (Sigma-Aldrich).

### Sample mounting

The coverslip was removed from the resin by dipping the block into liquid nitrogen. After localising the ROI at the blockface using the finder grid, the block was cut to fit in the ultramicrotome using a hacksaw, and trimmed with a razorblade to a frustum (truncated square pyramid) with height ∼2 mm and blockface of ∼500 μm×500 μm with the cell of interest located centrally (Fig. 1B). Parafilm™ was stretched over the blockface to reduce the risk of losing the frustum, which was then removed by cutting horizontally through the base (∼2 mm below the top surface) using a razorblade (Fig. 1C; Movie 1). Without touching the top surface, the frustum was dislodged from the parafilm and mounted onto an aluminium pin using conductive epoxy glue (ITW Chemtronics) so the blockface had a small tilt from horizontal (<5°) (Movie 2). The glue was brought up around the block sides for stability, but care was taken not to allow any of the glue onto the blockface. The glue was hardened at 60°C overnight, and sputter-coated with 2-nm Pt using a Q150R S sputter coater (Quorum Tech).

### SBF-SEM data collection

SBF-SEM data was collected using a 3View2XP (Gatan, Pleasanton, CA) attached to a Sigma VP SEM (Zeiss). The pin was loaded into the SBF-SEM. The knife was aligned parallel to the front edge of the block, and then to the highest corner or edge of the blockface with the SEM door open. The cell of interest was identified by adjusting the lighting to see through the Pt coating, using the light microscopy maps and images of the blockface acquired in the ultramicrotome during trimming for reference. A coarse approach was performed by cutting 100-nm sections until the Pt line was a few microns from the cell of interest. The chamber door was then closed and the SEM pumped to ∼5 Pa.

Using the BSE detector, an overview image of the whole blockface was acquired at 5 kV. At this voltage, charging effects were visible but it was possible to image through the Pt coat to the cells below, so that the final FOV could be determined. The final approach was performed at 50-nm slice thickness, cutting ten slices at a time and following the lateral progression of the Pt line towards the cell of interest.

The SEM imaging conditions for each dataset are presented in Table S2. For cutting analysis (Fig. 3A), 15-nm cuts were taken from a B cell sample containing 200-nm beads. Two clusters of beads in two separate areas in a cell were quantified. The number of cuts taken through each bead were counted and averaged to confirm slice thickness.

### TEM image acquisition

The SBF-SEM run was stopped once the structure of interest had been identified, using light microscopy maps and fluorescence microscopy images as a reference. The pin was removed from the SBF-SEM and placed in a UC7 ultramicrotome (Leica Microsystems). The cut face was aligned to the diamond knife as closely as possible. 100-nm thick sections were cut, collected on formvar-coated slot grids and imaged in a Tecnai G2 Spirit BioTwin TEM (FEI Company) using an Orius CCD camera (Gatan Inc.). Tilt series were acquired from ±70° using an Ultrascan CCD camera (Gatan Inc.) and Inspect 3D software (FEI Company). Tomograms were reconstructed using the back projection algorithm in IMOD software (Kremer et al., 1996).

### Image processing for 10-nm dataset from Vero E6 cells

To reduce noise and improve image contrast, the extracted image stack was batch processed using Adobe Photoshop to convert it into an 8-bit greyscale image stack, apply a 1 pixel Gaussian blur, and resharpen with an unsharp mask (60%, 20-pixel radius; then 50% 5-pixel radius). The image stack was then imported into Fiji (Schindelin et al., 2012), cropped to the area of interest, and contrast was normalised across all slices.

### Overlay of 3D light microscopy and 3D electron microscopy datasets

For 3D light microscopy-electron microscopy alignment, both image stacks were opened in Fiji. The light microscopy image stack was usually a multi-channel confocal *z*-stack from a single time-point. The native electron microscopy file format (Gatan Digital Micrograph ‘.dm4’ format) was opened using the Bio-Formats importer (http://www.openmicroscopy.org/site/support/bio-formats5.1/). If the image stack size was larger than the available memory it was opened as a virtual stack. This was rescaled in Fiji, ensuring that the rescaled electron microscopy pixel resolution was higher than the native light microscopy pixel resolution.

In the BigWarp plugin, the electron microscopy stack was set as ‘target’ and the light microscopy stack as ‘moving’ dataset. ‘Landmark mode’ was used to add at least four pairs of corresponding points within the light microscopy and electron microscopy data. Further points were added to refine the transform and correct for non-linear deformations caused by sample processing. The graphical user interface of BigWarp was particularly useful for these steps given that the transformation can be applied live, and even continuously adjusted by dragging a landmark. It was also useful for visualising feature correlation in orthogonal and oblique planes to fine-tune landmark placement; the electron microscopy dataset could be aligned to the plane of the Pt layer, which was coplanar with the coverslip in the light microscopy dataset (hence why it was advisable to acquire a full confocal stack from the coverslip upwards). It was important to choose points that were well separated in all three dimensions to minimise angular errors in the rotations. The final transformations were saved in BigWarp, which could then be applied to the full resolution data to produce the final overlay. The landmark table could be exported as a small textfile containing sufficient information to recreate the full alignment.

For manual segmentation and 3D model generation of Mtb and HIV-1 datasets, electron microscopy image stacks were converted to 8-bit greyscale tiff format images and a 0.5- or 1-pixel Gaussian blur applied, then aligned and manually segmented using Amira software (FEI Company). HIV budding profiles were manually placed using TrakEM2 (Schindelin et al., 2012), then their co-ordinates were extracted using a python script [courtesy of Albert Cardona, Howard Hughes Medical Institute (HHMI) at the Janelia Research Campus, VA] and imported into Amira for visualisation. For 3D rendering of the entotic cell dataset, we used the ClearVolume plugin in Fiji (http://fiji.sc/ClearVolume) (Royer et al., 2015). The SBF-SEM image was inverted and the Pt signal subtracted to aid visualisation in 3D.
